# Different strategy of hand choice after learning of constant and incremental dynamical perturbation in arm reaching

**DOI:** 10.3389/fnhum.2014.00092

**Published:** 2014-02-24

**Authors:** Chie Habagishi, Shoko Kasuga, Yohei Otaka, Meigen Liu, Junichi Ushiba

**Affiliations:** ^1^Center for Biosciences and Informatics, School of Fundamental Science and Technology, Graduate School of Science and Technology, Keio UniversityKanagawa, Japan; ^2^Department of Biosciences and Informatics, Faculty of Science and Technology, Keio UniversityKanagawa, Japan; ^3^Department of Rehabilitation Medicine, Keio University School of MedicineTokyo, Japan

**Keywords:** motor learning, decision making, force field, energy expenditure, hand bias

## Abstract

In daily life, we encounter situations where we must quickly decide which hand to use for a motor action. Here, we investigated whether the hand chosen for a motor action varied over a short timescale (i.e., hours) with changes in arm dynamics. Participants performed a reaching task in which they moved a specified hand to reach a target on a virtual reality display. During the task, a resistive viscous force field was abruptly applied to only the dominant hand (*DH*). To evaluate changes in hand choice caused by this perturbation, participants performed an interleaved choice test in which they could freely choose either hand for reaching. Furthermore, to investigate the effect of temporal changes on arm dynamics and hand choice, we exposed the same participants to another condition in which the force field was introduced gradually. When the abrupt force was applied, use of the perturbed hand significantly decreased and not changed during the training. In contrast, when the incremental force was applied, use of the perturbed hand gradually decreased as force increased. Surprisingly, even though the final amount of force was identical between the two conditions, hand choice was significantly biased toward the unperturbed hand in the gradual condition. These results suggest that time-varying changes in arm dynamics may have a greater influence on hand choice than the amplitude of the resistant force itself.

## Introduction

Humans can flexibly switch their active hand depending on a given action or task. Such decisions are easily made in most cases, and the process is immediate and automatic. For example, imagine the situation where we have to support a glass that is tipping over on a table. We may inherently use the hand that is closest to the glass, or we may use the dominant hand (*DH*) more if an action requires accuracy (Coelho et al., 2013). However, occasionally we face a situation where we cannot easily decide which hand is better to use, for example when an object is located about the same distance from both hands. Previous studies reported that when objects are located within a region of uncertainty the hand choice varies trial-by-trial (Mamolo et al., 2006; Stoloff et al., 2011). How our brain chooses a particular hand in such situations raises intriguing scientific questions.

The neural mechanisms of hand choice have been studied intensively. For example, a study showed that transcranial magnetic stimulation to the left posterior parietal cortex can modify hand choice (Oliveira et al., 2010). The stimulation led to an increase in left hand use when participants were asked to reach with one hand to a visual target appearing at a variable location on a semicircular array, indicating that the posterior parietal cortex is involved in hand choice. They also found that reaction time was prolonged as ambiguity about hand choice increased. Animal studies have revealed the neural mechanisms of hand choice more precisely. For example, Schieber (2000) showed that the ventral premotor cortex played a role in hand choice during reaching and grasping a food, using a pharmacological inactivation of the corresponding region.

Another recent study demonstrated that manipulating the virtual reward (i.e., the success of a motor action) in a reaching task could change hand choice in such ambiguous situations over a short timescale (Stoloff et al., 2011). In their experiments, participants were asked to reach a target randomly displayed at several locations on the computer monitor with their preferred hand. During the experiment, the reward of the task was manipulated by systematically decreasing the likelihood that the result of one trial would be judged as successful if a subject used their *DH* in the previous trial. The likelihood of success was manipulated by changing the radius of virtual target regions while maintaining their visual size. When exposed to such an environment, the *DH* was used less frequently even though the subjects had a minimal awareness that the likelihood of reward was manipulated. The behavioral data were fit to a mathematical model based on reinforcement learning and the reward parameter was maximized by a minimal error rate. This result implies that the reinforcement history of motor performances affected the hand choice, even over a short timescale.

In addition to the reward of motor performance, the movement effort (force required to achieve a task) is also a large determinant for the balance of hand use. In the previous study (O'Sullivan et al., 2009), the authors showed that movement effort is considered more than movement variability by the neural motor control system, using an experiment in which participants had to share force production between the left and right fingers to match a goal force as accurately as possible. Considering their findings, it is assumed that not only the short-time manipulation of the reward of motor performance, but also the short-time manipulation of arm dynamics should also modify hand choice. Transient changes in arm dynamics occur in our daily life, such as when we are fatigued by exercise. How do those changes affect our hand choice?

In this study, we used protocols previously used in computational neurobiology studies of motor learning to gain a systematic understanding of how the processes of hand choice are modified by changes in arm dynamics in a short timescale. Specifically, we used reaching tasks with artificially introduced dynamic perturbations (Shadmehr and Wise, 2005). The difference between ordinary motor learning studies and the current study was that we used a velocity-dependent resistive force field instead of a velocity-dependent perpendicular force field, because the resistive force field more closely resembles natural situations than the perpendicular one.

Therefore, firstly the purposes of the current study were to determine: (1) whether changes in arm dynamics can modify hand choice over a short timescale, and (2) which change in the dynamic environment (i.e., amplitude of the dynamic change or time consistency) has a greater effect on the use of the perturbed hand. To answer the latter question, we used two kinds of dynamical perturbation to the movement environment in the current study; the abrupt and the gradual perturbations. The reason why we focused on these two conditions was that a number of studies have indicated that the adaptation mechanisms of the gradual and abrupt perturbations are substantially different. For example, in visuomotor and force-field adaptation tasks, the adaptation to a gradual perturbation exhibited better retention (Kagerer et al., 1997; Michel et al., 2007; Huang and Shadmehr, 2009), and gradual and abrupt perturbation led to a distinct pattern of generalization (Malfait and Ostry, 2004; Michel et al., 2007; Kluzik et al., 2008). In addition, the learning of gradual and abrupt forces may be mediated by different neural substrates (Criscimagna-Hemminger et al., 2010; Schlerf et al., 2012; Orban de Xivry et al., 2013). Finally, unlike the manipulation of the reward as changes in the success rate of motor performance (Stoloff et al., 2011), our manipulation of arm dynamics may not necessarily reinforce a specific behavior. Therefore, we also expected to clarify mechanisms other than reinforcement learning that could result in hand choice changes.

## Materials and methods

### Ethics statement

This study was conducted according to the Declaration of Helsinki. The experimental procedures were approved by the ethical committees of the Faculty of Science and Technology, Keio University, and Keio University School of Medicine. Written informed consent was obtained from all participants prior to the experiments.

### Participants

Eighteen neurologically normal participants (5 women and 13 men, aged 21–34 years) participated in the experiments. We excluded data from 2 participants because a ceiling effect was observed in the data (all targets were selected by *DH*) for one participant, and the other one did not follow the instruction of the reaching hand. Thus data from 16 participants were included in the analyses. Three participants were left-handed and 13 participants were right-handed [laterality quotient (LQ) = 57.3 ± 15.6, expressed as the mean ± standard error (SE)], as assessed by the Edinburgh Handedness Inventory (Oldfield, 1971). All participants were naïve to the purpose of the experiments. All participants participated in both of the experiments (abrupt and gradual conditions) described in the *Experiments* section in a crossover design (the order of experiments was counterbalanced across participants), with an interval separated by 1–46 days.

### Apparatus

**Figure 1:**
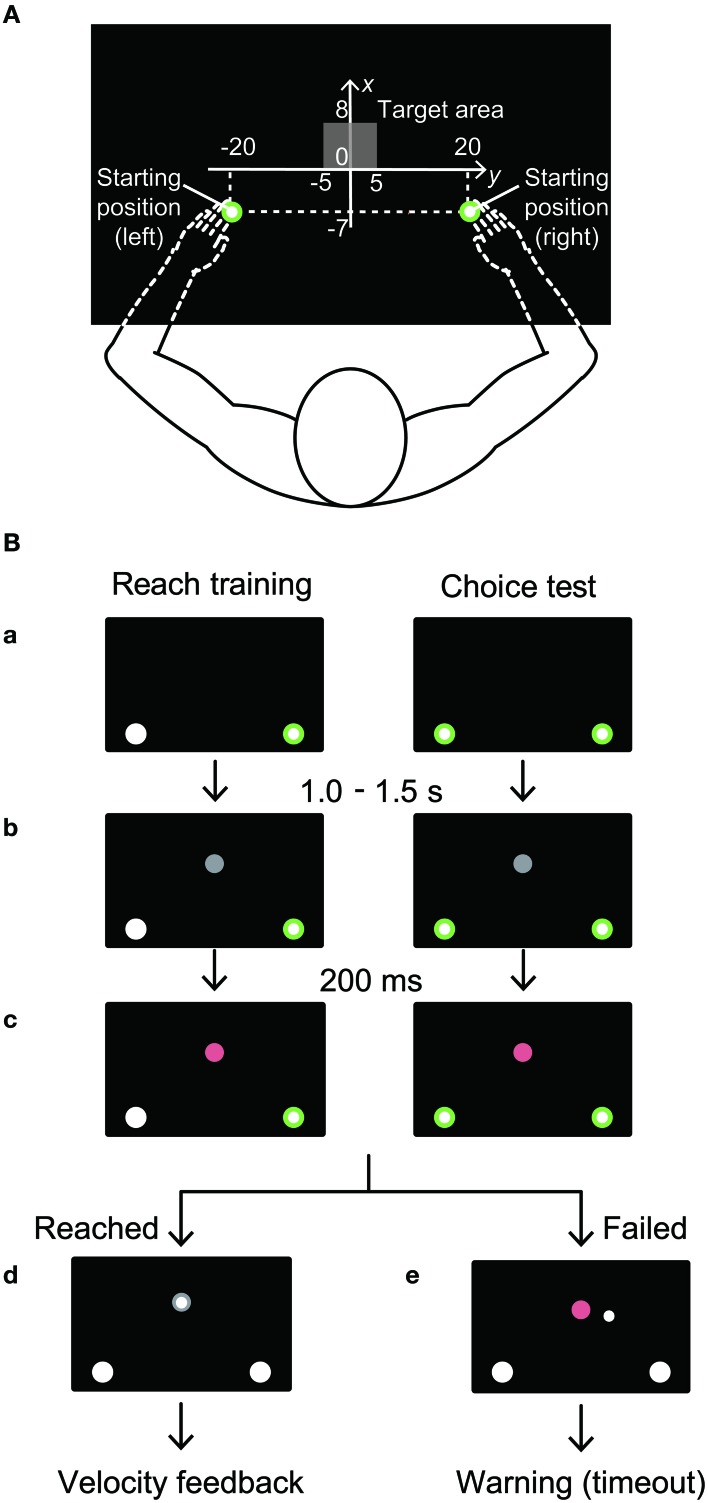
**(A)** Experimental procedures for the reach training and the choice test tasks. The origin of the frame of reference for the display was set at the midpoint of the fingertips when the elbow angle was 90° and the shoulder angle was 30° (full extension was 0°). The starting position for the left hand: (x, y) = (-20.0 cm, −7.0 cm). The starting position for the right hand: (x, y) = (20.0 cm, −7.0 cm). The target appeared randomly at one of 55 locations in the range of (x) = −5–5 cm, and (y) = 0–8 cm (gray shadow). The target appeared in every 1 cm on the *x*-axis and every 2 cm on the *y*-axis. **(B)** Task sequence. After participants held their hands in the starting positions for 1.0–1.5 s (randomized, a), a gray target appeared (b). As the “go” cue, the color of the target was changed to magenta 200 ms after the target appeared, and participants initiated their reaching movements (c). When participants stopped the reaching movement, the hands were automatically returned to the starting position, and feedback regarding the movement velocity was displayed on the screen (d). If neither hand moved within 200 ms after the “go” cue was presented, or did not reach the target 2.0 s after the movement onset, a warning message (“Timeout”) was displayed (e).

The experiments were performed with a bilateral robotic exoskeleton (KINARM Exoskeleton, BKIN Technologies, Canada; Scott, 1999). The robotic device supports the arms, forearms, and hands against gravity and permits only flexion and extension of the shoulder and elbow. The participants sat on a straight-backed chair with their arms abducted in the horizontal plane. We set the angle of abduction so that the arm, forearm, and hands were in the same plane as the shoulder (~80°). The arms, forearms, and hands were supported by plastic arm troughs that were attached to an adjustable 4-bar linkage. The tips of the participants' index fingers were presented to the participants (1.0 cm diameter, white circle) in a horizontal plane (72 × 35 cm) above the arms via a display constructed from an overhead projector and a semitransparent mirror (Figure 1A). A metal barrier under the mirror prevented the participants from directly seeing their arm. The participants controlled the cursor by performing reaching movements from a cue (2 cm diameter) positioned at the starting position, toward a target (2 cm diameter) displayed on the screen. The cursor and starting position were always visible. The origin of the frame of reference for the display was set at the midpoint of the fingertips when the elbow angle was 90° and the shoulder angle was 30° (full extension was 0°). The starting positions were located at (x, y) = −20 cm, −7 cm, for the left hand and (x, y) = 20 cm, −7 cm, for the right hand. The target appeared randomly at one of 55 locations in the range of (x) = −5 to 5 cm, and (y) = 0 to 8 cm at even intervals (Figure 1A). The position and velocity of the arms were initially A/D converted at 1.129 kHz, and then re-sampled and recorded at 1 kHz for offline analysis.

### General procedure

The experiment was designed to examine how hand choice was influenced when the dynamics of the dominant arm were changed by an externally applied force field. Participants were instructed to move either hand from the starting position to the target in a straight trajectory. At the start of the trial, participants were required to move the cursors into the starting positions (Figure 1B). After participants maintained the cursor at the starting position for 1.0–1.5 s, a gray target was presented. After 200 ms, the target's color changed from gray to magenta, which was the “go” cue. When participants completed the movement, the hand was automatically returned to the starting position. If the movement time between the movement onset and the target reach was above (“fast”) or below (“slow”) the given range, a warning message was presented on the screen to remind the participant to move with a constant speed. If the movement time was appropriate, the message “good” was presented. Based on the minimum jerk theory (Flash and Hogan, 1985), we used five time ranges that were dependent on the vertical position of the target, so that the peak velocity remained within 705.0 ± 70.5 mm/s when the participants reached to the central target of each vertical level. Movement onset was defined as the time point when the hand velocity first exceeded 5% of the estimated peak velocity. Movement offset was defined as the time point when the hand velocity first dropped below 5% of the estimated peak velocity after the time when the peak velocity of each trial was detected. If neither hand began moving within 200 ms of the “go” cue (i.e., 400 ms after the target appearance), or stopped moving (i.e., movement offset was detected) within 2.0 s of movement onset, the warning message “timeout” was presented and the trial ended.

### Reach training and choice test

Participants performed the reach training and choice test tasks in an alternating order (Figure 1B). In the reach training, participants were instructed to move either their right or left hand when the starting position turned green to the instructed target. In each task block, participants reached to all 55 targets with each hand, and therefore performed 110 trials in each block. Both the instructions for the reaching hand and the 55 targets were presented randomly. All trials where movement offset of instructed hand was detected within 2.0 s of movement onset were included in the subsequent offline analyses of the reach training. The percentage of the trials included was 93.5%. In the choice test, we identified which hand the participant preferred to use to reach toward a target. Participants were free to choose which hand they used. Participants reached to the same 55 randomly presented targets used in the reach training task. The choice test was performed five times (i.e., 275 trials for each block). All trials where movement offset of either hand was detected within 2.0 s of movement onset were included in the subsequent offline analyses of the choice test. The percentage of the trials included was 100.0%. Prior to the experiment, participants practiced both the reach training and choice test without any external perturbation.

**Figure 2:**
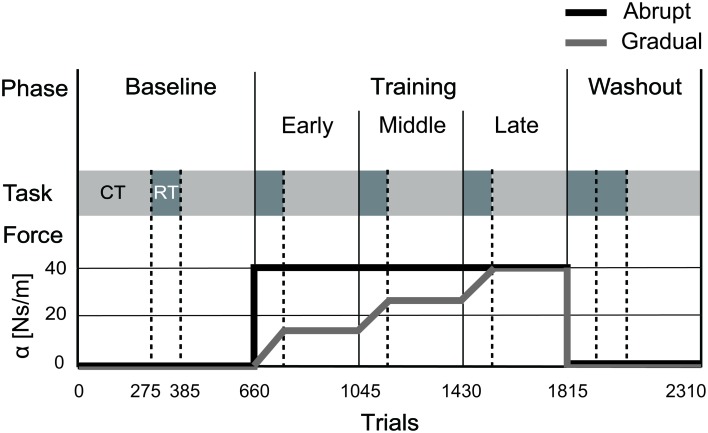
**Training Schedule. Upper** row: the experiments consisted of 3 different phases: the baseline, training, and washout phases. **Middle** row: each phase consisted of the choice test (CT; light gray) and the reach training (RT; dark gray). **Lower** row: The force field was applied either abruptly (black line) or gradually (gray line). All participants performed the task under both conditions in a crossover design.

The experiments included 3 different phases, with each phase consisting of the reach training and the choice test (Figure 2). First, in the baseline phase, we investigated whether the number of null trials performed (i.e., trials without the force field) influenced hand choice. In this phase, the choice test was performed twice, before and after the reach training. Next, we examined how hand choice was influenced by changes in arm dynamics. Therefore, in the second phase (i.e., training phase), a velocity-dependent resistive force field (Equation 1) was applied to only the *DH* in the training phase.

(1) $$F(x,\;y)=-\alpha \vec{v}$$

We applied the force to the *DH* because a previous study (Stoloff et al., 2011) reported that people were more likely to use their *DH* after an error in one trial, regardless of whether that error was produced with the dominant or non-dominant hand. Therefore, if we applied the force to the non-dominant hand (*NDH*), we would not be able to dissociate the effect of this natural tendency from changes in hand choice resulting from the dynamic perturbations.

In the training phase, a combination of the 2 tasks, reach training and the choice test, were repeated 3 times. Therefore, we divided the training phase into 3 blocks: “early training block,” “middle training block,” and “late training block.”

Finally, the washout phase to detect the aftereffects of force field learning on hand choice was performed. In this phase, participants first performed the reach training in the null field twice. After the 2 sets of reach training, they performed the choice test.

Each choice test (275 trials) took about 20–25 min, and each set of reach training (110 trials) took about 8–9 min. Ten minutes breaks were enforced after participants performed the baseline phase and each training block.

### Abrupt and gradual conditions

To investigate whether changes in a given property affect hand choice, we either abruptly or gradually applied the force field to the participants' movements (Figure 2). In the abrupt condition, the maximum amount of force (α = 41.25 Ns/m) was abruptly applied from the first trial of the early training block, and was maintained throughout the training phase. In addition, to investigate the effect of temporal changes in the dynamic environment on hand choice, participants performed the task in another condition (the gradual condition). In the gradual condition, the amount of force was increased gradually in each trial during the reach training (+0.25 Ns/m per trial). In the choice test, during the training phase, force was kept at a constant level, and was equal to that used in the last trial of the corresponding reach training (α = 13.75, 27.50, 41.25 Ns/m for the choice test in the early, middle, and late training blocks, respectively).

### Questionnaires

In both the abrupt and gradual conditions, at the end of the experiments, we performed a semi-structured interview about participants' awareness of the perturbation. The questions were as follows:

1. Did you notice that the external force was applied to your hands? (If Yes)
2. Was the force applied to both hands or to only one hand?
3. When did you feel the force?
4. Were there any changes in the force level during the experiment? (If Yes)
5. Did you feel any difference in force levels between the reach training and the choice test?
6. Did you feel any trial-by-trial difference in force levels?

### Data analysis

Data recorded in the reach training was analyzed by calculating the movement kinematics (movement time, peak velocity, and movement accuracy). Movement time was defined as the time from movement onset to offset. Peak velocity was defined as the maximum movement velocity between movement onset and offset. Movement accuracy was defined as the squared errors (i.e., squared distance from the target) at movement offset.

Data recorded in the choice test were analyzed by calculating the hand bias (*HB*) and reaction time (i.e., time between the go cue and movement onset) in the choice test of each phase.

*HB* represents the preference for using one hand in a given workspace on the display. *HB* was calculated by subtracting the total number of times the *NDH* was used from the number of times the *DH* was used, and dividing the result by the total number of reaches performed.

(2) HB=(DH−NDH)/(DH+NDH)

For example, when *HB* was positive, the participant used the *DH* more than the *NDH*. *HB* can take values from −1 to 1, with a value of 1 indicating the participant used only the *DH*, while a value of −1 indicated the participant used only the *NDH*. We aimed to use this parameter to quantify the effect of a perturbation on the *DH*. Both right- and left-handed participants were included in the study; therefore, we used *DH* and *NDH* to analyze the data from all participants together. Prior to the analyses, we confirmed the validity of including the left-handed participants in the study by checking the baseline *HB* in the left-handed participants. We found that all but one condition in one participant was within the 95% confidence interval of the *HB* calculated from the *HB*s in right-handed participants.

### Statistics

The data are expressed as the mean ± SE. A Two-Way repeated measures analysis of variance (ANOVA) was used to investigate differences in *HB* between the two baseline choice tests and conditions, the reaction time differences between the choice tests and conditions, and differences in movement kinematics for the following comparisons: (i) hand × condition for the baseline data, (ii) hand × block for each condition, and (iii) condition × block for each hand. A Two-Way factorial ANOVA (order × hand) was performed to detect the effect of experimental order on the kinematics in the baseline phase. For the block comparisons, we used data from the baseline phase, early training block, middle training block, late training block, and washout phase. Bonferroni multiple comparison tests were used for the *post-hoc* test in the Two-Way ANOVA of movement kinematics, and for the comparisons of *HB* between blocks. Paired *t*-tests were used to detect the effect of experimental order on *HB* in the first choice test of each participant, differences in *HB* changes between the baseline phase and the last training block between the two force conditions. The significance threshold was set at *P* < 0.05.

## Results

### Hand preference in the baseline phase

**Figure 3:**
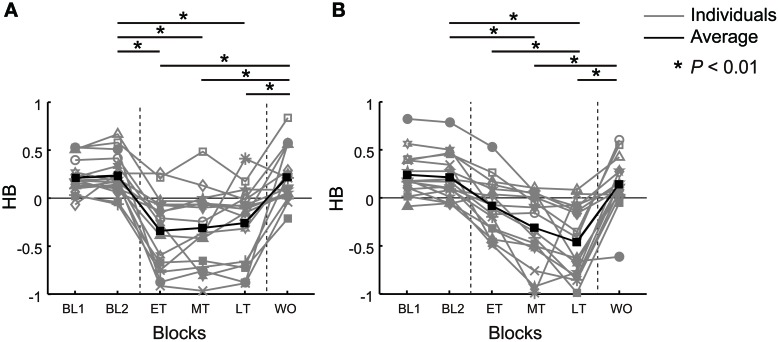
***HB* in the abrupt and gradual condition from the baseline blocks (BL1, BL2), early training block (ET), middle training block (MT), late training block (LT), and the washout block (WO).** Gray lines indicate *HB* of individual participants and black lines indicate the average *HB*. The horizontal lines with an asterisk above the panel represent significant differences between blocks (*P* < 0.01). **(A)** *HB* for all participants in the abrupt condition. **(B)** *HB* for all participants in the gradual condition.

In the baseline phase of the current experiments, the choice test in the null field showed a similar hand preference tendency for both conditions (Figure 3). We examined whether *HB* in the baseline phase was greater than zero (i.e., hand use was biased toward the *DH*) by calculating the lower bound of the 99% confidence interval of the sample mean for the first and second choice tests. *HB* in the baseline phase was significantly biased toward the *DH* (abrupt, mean ± SE of *HB* = 0.22 ± 0.05 in the first choice test and 0.24 ± 0.05 in the second choice test; gradual, *HB* = 0.24 ± 0.06 in the first choice test and 0.21 ± 0.06 in the second choice test).

We confirmed that there was no effect of experimental order on the baseline choice test. A paired *t*-test indicated that there was no difference in *HB* [*t*_(15)_ = 0.06, *P* = 0.95] between the first choice test in the baseline phase in the first condition (e.g., the abrupt condition) and the first choice test in the baseline phase in the second condition (e.g., the gradual condition, and vice versa).

There was no significant difference in *HB* between the two choice tests [*F*_(1, 60)_ = 0.01, *P* = 0.94] in the baseline phase or between the conditions [*F*_(1, 60)_ = 0.00, *P* = 0.98]. These results indicate that the repetition of the baseline choice test did in general not influence the hand choice. For simplicity, we only used the results of the second choice test as baseline in subsequent analyses. We did not pool the two baseline choice tests in order to equalize the number of trials between the baseline phase and each training block, and the baseline phase and the washout phase.

### Hand preference in the abrupt condition

In the abrupt condition, all participants reported that they noticed an abrupt force increment on the *DH* in the early training block. *HB* abruptly decreased in the early training block for all participants. Figure 3A represents the *HB* in all participants for the abrupt condition. In each training block, *HB* decreased significantly compared to the baseline block (baseline, *HB* = 0.24 ± 0.05; early training, *HB* = −0.34 ± 0.09, *P* < 0.01; middle training, *HB* = −0.31 ± 0.10, *P* < 0.01; late training, *HB* = −0.26 ± 0.10, *P* < 0.01, Bonferroni corrected). *HB* was not changed during the training blocks (difference between early and middle training, *P* = 1.00; early and late training, *P* = 1.00; middle and late training, *P* = 1.00, Bonferroni corrected).

### Hand preference in the gradual condition

In the gradual condition, all the participants noticed the introduction of the force field on the *DH* in the early training block. However, they did not notice the trial-by-trial increment in force, though they reported feeling that their *DH* was heavier in one block than in a previous block. There were no reported differences regarding whether the abrupt or gradual condition had been performed first. The decrease in *DH* use during the training phase was seen in almost all participants. Figure 3B presents the *HB* for the gradual condition. In the middle and the late training blocks, *HB* decreased significantly from the baseline (baseline, *HB* = 0.21 ± 0.06; middle training, *HB* = −0.31 ± 0.09, *P* < 0.01; late training, *HB* = −0.46 ± 0.09, *P* < 0.01, Bonferroni corrected). In addition, *HB* gradually decreased during the training phase. A significant decrement was noted between the early and the late training blocks (early training, *HB* = −0.08 ± 0.07, *P* < 0.01, Bonferroni corrected).

### Difference in *HB* in the training phase between conditions

**Figure 4:**
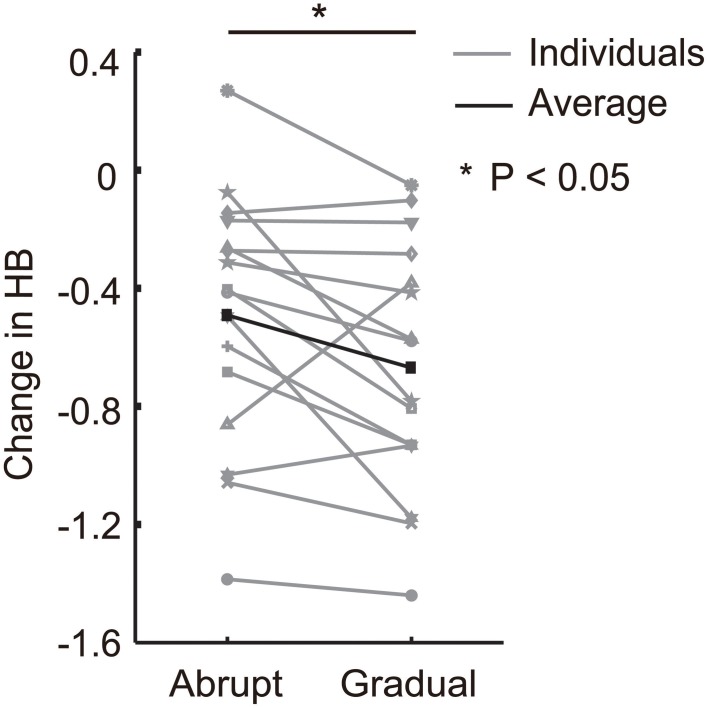
**Changes in *HB* observed from the baseline to the late training blocks during the abrupt and gradual conditions.** Gray lines represent the results for each participant. Black line represents the average change in *HB* for all participants. An asterisk indicate significant differences between conditions (*P* < 0.05).

To detect the difference in *HB* between the conditions, we compared changes in *HB* from the baseline block to the late training block in both conditions. A paired *t*-test detected significant differences between the conditions [*t*_(15)_ = −2.44, *P* < 0.05; Figure 4]. It is noteworthy that although the amount of force applied was identical in the two conditions during the late training block, decrease in *HB* from the baseline was significantly larger in the gradual condition than the abrupt condition. We also directly compared *HB* in the late learning block between the conditions, and found that *HB* in the gradual condition was significantly biased toward the *NDH* than the abrupt condition [*t*_(16)_ = −2.77, *P* < 0.05].

### Kinematic differences between the hands in the abrupt and gradual conditions

**Figure 5:**
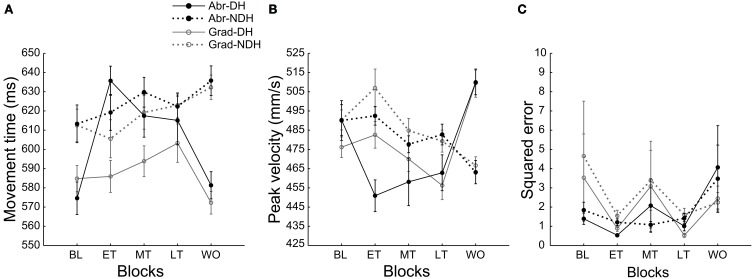
**Movement kinematics from the baseline block (BL), early training block (ET), middle training block (MT), late training block (LT), and the washout block (WO).** Movement time **(A)**, Peak movement velocity **(B)**, and Accuracy of movement **(C)** are shown in each panel. Filled circles with black lines indicate the abrupt condition (Abr), and open circles with gray lines indicate the gradual condition (Grad). Solid lines indicate the dominant hand (-DH), and dotted lines indicate the non-dominant hand (-NDH).

All kinematics data are provided in the Tables 1–3 and the Figure 5. At first we confirmed that there was no baseline difference between the conditions in all the kinematic parameters [movement time, *F*_(1, 60)_ = 0.30, *P* = 0.59; peak velocity, *F*_(1, 60)_ = 0.78, *P* = 0.38; movement accuracy, *F*_(1, 60)_ = 1.70, *P* = 0.20]. Baseline difference between the hands was observed only in movement time [*F*_(1, 60)_ = 14.6, *P* < 0.01], indicating that movement time was shorter in the *DH*. Difference between the hands was not detected in peak velocity [*F*_(1, 60)_ = 0.82, *P* = 0.37] or movement accuracy [*F*_(1, 60)_ = 0.17, *P* = 0.68]. We also investigated whether there was any effect of experimental order; however, no difference was detected in all the parameters [movement time, *F*_(1, 60)_ = 1.35, *P* = 0.25; peak velocity, *F*_(1, 60)_ = 0.55, *P* = 0.46; movement accuracy, *F*_(1, 60)_ = 1.03, *P* = 0.31].

**Table 1:** **Movement time (ms) from the baseline block (BL), early training block (ET), middle training block (MT), late training block (LT), and the washout block (WO)**.

	**BL**	**ET**	**MT**	**LT**	**WO**
Abr-DH	574.6±8.7	635.7±7.8	617.5±11.5	615.1±13.1	581.3±7.3
Abr-NDH	613.3±10.1	619.3±9.6	629.8±8.0	622.3±6.8	635.7±7.9
Grad-DH	584.8±7.1	585.9±8.6	593.9±8.3	603.2±10.3	572.3±6.1
Grad-NDH	612.6±8.6	605.5±10.2	619.2±9.0	623.0±6.7	632.3±6.6

Abr-DH, the dominant hand in the abrupt condition; Abr-NDH, the non-dominant hand in the abrupt condition; Grad-DH, the dominant hand in the gradual condition; Grad-NDH, the non-dominant hand in the gradual condition.

**Table 2:** **Peak movement velocity (mm/s) from the baseline block (BL), early training block (ET), middle training block (MT), late training block (LT), and the washout block (WO)**.

	**BL**	**ET**	**MT**	**LT**	**WO**
Abr-DH	490.2±8.3	450.9±8.5	458.1±12.8	462.8±9.6	509.9±6.7
Abr-NDH	490.1±10.7	492.5±5.0	477.7±4.5	482.7±5.6	463.2±6.3
Grad-DH	476.2±5.5	482.6±7.1	470.0±6.7	456.3±7.7	509.5±7.6
Grad-NDH	490.3±5.4	507.0±10.2	484.8±6.6	479.3±5.9	466.6±4.7

Abr-DH, the dominant hand in the abrupt condition; Abr-NDH, the non-dominant hand in the abrupt condition; Grad-DH, the dominant hand in the gradual condition; Grad-NDH, the non-dominant hand in the gradual condition.

**Table 3:** **Squared errors (an index of movement accuracy) from the baseline block (BL), early training block (ET), middle training block (MT), late training block (LT), and the washout block (WO)**.

	**BL**	**ET**	**MT**	**LT**	**WO**
Abr-DH	1.392±0.303	0.534±0.052	2.076±1.428	1.010±0.256	4.066±2.235
Abr-NDH	1.842±0.424	1.210±0.182	1.082±0.174	1.418±0.264	3.481±1.797
Grad-DH	3.531±2.343	0.891±0.156	3.080±2.414	0.530±0.111	2.445±0.663
Grad-NDH	4.652±2.941	1.549±0.299	3.393±1.608	1.599±0.347	2.238±0.539

Abr-DH, the dominant hand in the abrupt condition; Abr-NDH, the non-dominant hand in the abrupt condition; Grad-DH, the dominant hand in the gradual condition; Grad-NDH, the non-dominant hand in the gradual condition.

**Figure 6:**
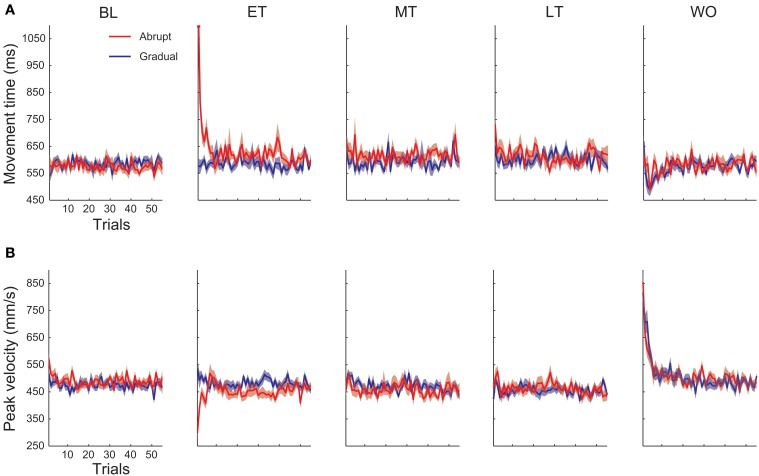
**Trial-by-trial changes in movement time (A) and peak velocity (B) from the baseline block (BL), early training block (ET), middle training block (MT), late training block (LT), and the washout block (WO).** Red lines indicate the abrupt condition and blue lines indicate the gradual condition. The shaded colored region represents the SE.

Next, we investigated whether there were any differences or changes in movement kinematics during the training phase between the hands for each condition. For the abrupt condition, a Two-Way repeated measures ANOVA (hand × block) detected a significant main effect of hand [*F*_(1, 135)_ = 10.3, *P* < 0.01], block [*F*_(4, 135)_ = 4.07, *P* < 0.01] and an interaction effect [*F*_(4, 135)_ = 4.31, *P* < 0.01] for movement time (Figure 5A). A *post-hoc* test showed significant increase in movement time from the baseline to each training block (early training, *P* < 0.01; middle and late training, *P* < 0.05, Bonferroni corrected) only for the *DH*. Figure 6 indicates trial-by-trial changes in movement time and peak velocity for the *DH* from the baseline to the washout blocks. The figure shows a sudden increase in movement time for the *DH* at the beginning of the early training block (Figure 6A). Difference between the hands was not significant in any training blocks; however, we need to consider the baseline difference. For peak velocity (Figure 5B), there was a main effect of block [*F*_(4, 135)_ = 3.54, *P* < 0.01] and an interaction effect [*F*_(4, 135)_ = 10.2, *P* < 0.01]. There was a significant decrease from the baseline to the early and middle training block only for the *DH* (early training, *P* < 0.01; middle training, *P* < 0.05, Bonferroni corrected). Figure 6B shows a sudden decrease in peak velocity for the *DH* at the beginning of the early training block. Although no main effect of hand was detected for peak velocity, a *post-hoc* test showed that peak velocity was significantly slower for the *DH* than for the *NDH* in the early training block (*P* < 0.01, Bonferroni corrected). A significant main effect of block was detected for movement accuracy [Figure 5C; *F*_(4, 135)_ = 2.75, *P* < 0.05]; however, a *post-hoc* test detected no significant difference between the blocks for either hand.

For the gradual condition, there was a significant main effect of hand [*F*_(1, 135)_ = 35.9, *P* < 0.01] for movement time. A *post-hoc* test showed movement time was significantly shorter for the *DH* in the middle training block (*P* < 0.05, Bonferroni corrected); however, we need to consider the baseline difference. For peak velocity, a significant main effect of hand [*F*_(1, 135)_ = 4.28,*P* < 0.05], block [*F*_(4, 135)_ = 8.05, *P* < 0.01], and an interaction effect [*F*_(4, 135)_ = 15.0, *P* < 0.01] were detected. A *post-hoc* test showed peak velocity was significantly slower for the *DH* in each training block (early training, *P* < 0.01; middle training, *P* < 0.05; late training, *P* < 0.01, Bonferroni corrected). There was significant decrease in velocity from the early to the late training block for the *DH* (*P* < 0.01, Bonferroni corrected), and the early to the middle training block (*P* < 0.05, Bonferroni corrected) and the early to the late training block (*P* < 0.01, Bonferroni corrected) for the *NDH*. For movement accuracy, no difference between the hands or the blocks was detected [effect of hand, *F*_(1, 135)_ = 0.45, *P* = 0.50; effect of block, *F*_(1, 135)_ = 1.74, *P* = 0.14].

### Difference in the kinematics of each hand between conditions

Next, we investigated whether there was any difference in the movement kinematics between the conditions during the training phase for each hand. For the *DH*, a Two-Way repeated measures ANOVA (condition × block) detected a significant main effect of condition [*F*_(1, 135)_ = 16.3, *P* < 0.01], and an interaction effect [*F*_(4, 135)_ = 5.61, *P* < 0.01] for movement time. A *post-hoc* test showed that movement time was significantly longer for the abrupt condition in the early and the middle training block (early training, *P* < 0.01; middle training, *P* < 0.05, Bonferroni corrected). There was no significant main effect of condition for peak velocity [*F*_(1, 135)_ = 0.93, *P* = 0.34], but an interaction effect was detected [*F*_(4, 135)_ = 2.95, *P* < 0.05]. A *post-hoc* test showed that peak velocity was significantly slower for the abrupt condition in the early training block (*P* < 0.01, Bonferroni corrected). There was no significant main effect of condition for movement accuracy [*F*_(1, 135)_ = 0.10, *P* = 0.75].

For the *NDH*, the difference between the conditions was not detected in any kinematic parameters [movement time, *F*_(1, 135)_ = 2.81, *P* = 0.96; peak velocity, *F*_(1, 135)_ = 1.73, *P* = 0.19; movement accuracy, *F*_(1, 135)_ = 1.36, *P* = 0.25].

### Changes in reaction time

We compared the reaction time in the choice tests for the abrupt (baseline, 151.7 ± 7.0 ms; early training, 151.0 ± 7.5 ms; middle training, 148.5 ± 6.5 ms; late training, 146.0 ± 6.6 ms; washout, 148.3 ± 7.2 ms) and the gradual (baseline, 154.8 ± 5.6 ms; early training, 156.6 ± 6.1 ms; middle training, 145.9 ± 6.8 ms; late training, 143.2 ± 6.4 ms; washout, 148.7 ± 6.4 ms) conditions. A Two-Way repeated measures ANOVA (condition × block) detected no significant main effect of condition [*F*_(1, 150)_ = 0.03, *P* = 0.86] or block [*F*_(4, 80)_ = 0.72, *P* = 0.58].

### Aftereffects in the washout phase

To investigate whether there was any aftereffect of the resistive force field, we first compared the *HB* of the choice tests in the baseline and the washout blocks (abrupt, baseline, *HB* = 0.24 ± 0.05, washout, *HB* = 0.22 ± 0.07; gradual, baseline, *HB* = 0.21 ± 0.06, washout, *HB* = 0.14 ± 0.07). However, a Two-Way repeated measures ANOVA (condition × block) did not detect a difference between the conditions [*F*_(1, 48)_ = 0.71, *P* = 0.40] or the blocks [*F*_(1, 48)_ = 0.44, *P* = 0.50].

Second, to investigate the aftereffects from another aspect, we also compared the movement kinematics of reach training in the baseline and washout blocks. Although the participants performed a set of reach training twice in the washout phase (Figure 2), we only analyzed the data from the first block because kinematic aftereffects are usually transient and dissipate quickly. For the abrupt condition, all kinematic parameters were not different between the baseline and the washout blocks either for the dominant (movement time, *P* = 1.00; peak velocity, *P* = 0.62; movement accuracy, *P* = 0.53, Bonferroni corrected) and the non-dominant (movement time, *P* = 0.96; peak velocity, *P* = 0.11; movement accuracy, *P* = 1.00, Bonferroni corrected) hand.

For the gradual condition, movement time was not different between the baseline and the washout blocks both for the dominant (*P* = 1.00, Bonferroni corrected) and the *NDH* (*P* = 0.86, Bonferroni corrected). Peak velocity was significantly faster in the washout block for the *DH* (*P* < 0.01, Bonferroni corrected), while significantly slower in the washout block for the *NDH* (*P* < 0.05, Bonferroni corrected). Movement accuracy was not different between the baseline and the washout blocks either for the dominant (*P* = 1.00, Bonferroni corrected) and the non-dominant (*P* = 1.00, Bonferroni corrected) hand.

For both conditions, movement time was significantly shorter for the *DH* than the *NDH* in the washout block (abrupt, *P* < 0.01; gradual, *P* < 0.01, Bonferroni corrected); however, we need to consider that there was a significant baseline difference between the hands in movement time. Peak velocity was significantly faster for the *DH* than the *NDH* in the washout block (abrupt, *P* < 0.01; gradual, *P* < 0.01, Bonferroni corrected). There was no difference between the conditions in any kinematic parameters in the washout block.

Although there was no significant aftereffects at the block level in most cases, in the initial period of the washout block we found clear decrease in movement time (mean ± SE of initial 10 trials, abrupt, 563.1 ± 9.3 ms; gradual, 543.9 ± 10.7 ms; Figure 6A) and increase in peak velocity (mean ± SE of initial 10 trials, abrupt, 564.4 ± 24.3 mm/s; gradual, 597.1 ± 17.3 mm/s; Figure 6B) in the *DH*. When comparing the initial 10 trials of the washout block with the baseline block, a Two-Way repeated measures ANOVA (condition × block) detected a significant main effect of block [movement time, *F*_(1, 60)_ = 8.42, *P* < 0.01; peak velocity, *F*_(1, 60)_ = 38.9, *P* < 0.01], but no main effect of condition [movement time, *F*_(1, 60)_ = 0.25, *P* = 0.62; peak velocity, *F*_(1, 60)_ = 0.38, *P* = 0.54].

## Discussion

In the present paper, we aimed to understand how hand choice was influenced by short-term changes in arm dynamics by exposing participants to a novel dynamic environment. To address this issue, we trained participants to reach toward randomly appearing targets with the instructed hand, while we applied a viscous resistant force field to only the *DH*. We then intermittently tested the hand choice under the condition where participants could freely choose which hand they used to perform the reaching movement. Furthermore, to clarify what property of the arm dynamic changes had a stronger effect on hand choice, we introduced the force field either abruptly or gradually. We found that a gradual change had a greater effect on the decreased use of the perturbed hand than an abrupt change. The movement kinematics changed differently between the hands or the conditions during the experiment.

### Learning mechanism of the velocity-dependent resistive force field

First, our participants tended to use their *DH* more than their *NDH* in the baseline block, which has been reported in previous studies (Fisk and Goodale, 1985; Carey et al., 1996). However, when the dynamic perturbation was introduced, such tendencies disappeared.

In the abrupt condition, we observed that most participants showed a rapid decrease in the use of the perturbed hand in the early training block, and it did not change during the training phase or gradually increased again in the later training blocks in some participants (Figure 3A). In the gradual condition, *HB* gradually decreased for most participants as the force increased (Figure 3B). In addition, even though the amount of applied force was the same between the two conditions, *HB* was significantly lower in the gradual condition than in the abrupt condition in the late training block (Figure 4). This was surprising because we had naïvely assumed that the gradual force increase would be more easily compensated, and thus would not have an influence on *HB*. In fact, in previous studies where a force field was applied either abruptly or gradually, there were less trial-by-trial errors in the gradual condition than in the abrupt condition (Kagerer et al., 1997).

However, unlike the previous experiments where velocity-dependent forces were applied in the perpendicular to the movement direction, in our experiment, the resistive force field did not result in any directional errors. Therefore, in the current study, we do not expect adaptation to the force field by the development of an internal model through error-based learning (Wolpert et al., 1998; Kawato, 1999; Bastian, 2006), as was assumed in the studies using a perpendicular force field. In fact, movement accuracy did not show any erroneous control of movement in the early training block, or the systematic improvement of motor performance during the training phase (Figure 5). Instead, if we consider the shift in *HB* as a learning effect for assessing the levels of learning in our experiment, our results may not contradict the previous findings showing that the gradual perturbation resulted in better learning.

Such an interpretation that regards the shift in *HB* as a learning effect is still in contrast to previous findings using a velocity-dependent perpendicular force field or a visuomotor transformation. That is, we did not find any aftereffects in *HB* in either condition, while previous studies reported a difference in the aftereffects between the conditions (e.g., exhibited larger aftereffects or better retention in the gradual condition; Kagerer et al., 1997; Michel et al., 2007; Huang and Shadmehr, 2009). Therefore, we suggest that the learning of the resistive force field reflected in the shift in *HB* appears only when the perturbation exists.

The aftereffects of reaching under the resistive force field appeared in another aspect. Interestingly, we found a significant decrease in movement time and an increase in peak velocity in the initial phase of the washout blocks for both the abrupt and the gradual conditions. However, there was no difference between the conditions, while previous studies reported the aftereffects appeared differently between the abrupt and the gradual perturbations (Kagerer et al., 1997; Michel et al., 2007; Huang and Shadmehr, 2009). Therefore, we again suggest that the learning mechanism of the resistive force field is different from the adaptation to the velocity-dependent perpendicular force field or the visuomotor transformation.

### Driving force of the changes in hand choice

One possibility for the driving force behind the changes in hand choice is the reinforcement learning process as shown by Stoloff et al. (2011). Recently, many studies have provided evidence of the importance of the reinforcement process in motor learning, which is to find a smaller variable or less effortful solution for reducing movement costs (Izawa and Shadmehr, 2011; Stoloff et al., 2011; Wolpert et al., 2011; Haith et al., 2012; Shmuelof et al., 2012). For example, reinforcement learning is considered to contribute to the action selection process in the motor learning system (Shadmehr and Krakauer, 2008; Izawa and Shadmehr, 2011). Therefore, this type of learning may also play a role in effector selection such as hand choice. However, because we did not explicitly manipulate reward in the present study, we should not strongly relate our results to the reinforcement learning process.

In fact, considering our movement kinematics results (Figure 5) there was no strong evidence that the use of the unperturbed hand was reinforced. First, movement time increased during the training phase for the *DH* in the abrupt condition, but did not change for the *NDH*. In the gradual condition, we did not find any change in movement time during the training phase for both hands. In addition, movement time was not different between the conditions in the late training block for both hands. According to the results suggesting a correlation between the vigor of movement and decreased movement time (Choi et al., 2014), if the use of the *NDH* was reinforced through increased movement vigor, movement time should decrease in the *NDH*. In addition, movement time for the *DH* should be shorter for the abrupt condition than for the gradual condition in the late training block, because *HB* in the abrupt condition was more biased toward the *DH* than the gradual condition. Second, previous studies also showed that peak velocity increased as the vigor of movement increased (Shadmehr et al., 2010; Choi et al., 2014). In the current study, peak velocity was faster for the *NDH* than the *DH* in many training blocks for each condition. However, there was no difference between the conditions in the late training block. Thus, this parameter also cannot fully explain the increased use of the *NDH*. Third, there was no difference in movement accuracy between the hands or the conditions, implying that there was no difference in the reward from motor performance. Therefore, we suggest that the changes in hand choice observed in the current experiment were mediated by mechanisms other than reinforcement learning. Several other possibilities are discussed below.

We suggest that the major driving force behind the changes in hand choice was the trial-by-trial changes in arm dynamics. We also expect that the energy expenditure (force required to make movements) is one of the factors that caused the reduced use of the *DH*. However, the latter effect was limited if the force was not consistent across trials. This is because despite the fact that *HB* was smaller for the gradual condition in the late learning block, the impulse of the applied load was larger in the abrupt condition than in the gradual condition, and the force level was the same in the late learning block in the two conditions. Although it was not statistically significant, reduced use of the *DH* in the abrupt condition gradually recovered during the training phase. This suggests that when the participants were exposed to the abrupt introduction of the constant resistive force field, they may have used some explicit strategy to increase the use of the perturbed hand regardless of the high-energy expenditure. Such a strategy may not be adopted if the force increased in a trial-by-trial manner (i.e., the force was variant across trials). These results lead to the assumption that if the participants continued to perform the task under the constant force field after the gradual perturbation, they would recover the use of their *DH*. Further investigation is needed to establish the characteristics of *HB* after exposure to the gradual condition.

Another possibility is perceptual changes. We cannot know in what process of hand choice from planning to motor output, choice was biased toward the *NDH* by the current experiment. It is possible that hand choice was not only changed by the physical load, but also by changes in spacial perception. That is, perception of the target locations slightly shifted to the non-dominant side during the experiment. Indeed, recently Hagura et al. (2013) reported that visual motion perception was modulated by external forces applied to the hand that was used to respond the perceived motion directions. Further study to clarify the driving force behind the changes in hand choice is expected.

Finally, a limitation of the current study is that the movement kinematics data inherited large standard errors (i.e., individual variability). In the future, more precise control of the experimental tasks and specific instructions to the participants should improve the quality of the data.

### Implication for clinical applications

Although we originally developed our task to perform basic neuroscience research on the mechanisms of hand choice, the reach training and choice test in the current study could be used in clinical practice. For example, patients with hemiparesis resulting from stroke often exhibit a behavioral state in which use of the affected hand decreases while use of the unaffected hand increases, which is called learned non-use (Sterr et al., 2002). Learned non-use persists even after the motor functions of the affected hand have recovered. Further, learned non-use can be harmful because it may prevent reorganization in the cortical representation of the affected hand after stroke (Liepert et al., 2000), or slow down the recovery of coordinated bilateral movements necessary for daily activities (Choi et al., 2014).

Recently, Han et al. (2013) developed a new Bilateral Arm Reaching Test (BART) for the assessment of learned non-use, which visualizes the spatial probability distribution of hand choice. Similar to BART, our choice test could also be used for the quantitative measurement of learned non-use. Moreover, we may be able to use the same task setting for a therapeutic intervention, by applying arbitrary forces to the hands during training. Appropriate manipulation of dynamic arm properties may regain the balance of hand choice during the training. Although the applicability is an open question, if we could facilitate the use of the affected hand by a gradual manipulation of arm dynamics during rehabilitation training, it may be able to induce use-dependent functional recoveries of the affected hand. A previous study reported that use-dependent learning, the mechanism changed movements to become more similar to the last movement, was different from error-based learning or reinforcement learning (Diedrichsen et al., 2010). Further studies are expected to investigate the relationship between behavioral changes we observed in the current experiments and use-dependent learning, and the effect of abrupt and gradual perturbation on use-dependent learning.

## Conclusion

In the current study, we found that changes in arm dynamics induced by a velocity-dependent resistive force field could modify hand choice in a short timescale. Hand choice modifications were different depending on how the resistive force was applied, rather than on the amount of force. A trial-by-trial increase in force resulted in a larger shift in hand bias at the end of learning than the abrupt and consistent increase in force. The movement kinematics showed that such a shift in hand bias could not be explained by reinforcement learning, suggesting that mechanisms besides reinforcement learning could modify hand choice.

## Author contributions

Chie Habagishi and Shoko Kasuga equally contributed to this work. Chie Habagishi, Shoko Kasuga, and Junichi Ushiba designed the experiments. Chie Habagishi and Shoko Kasuga performed research. Chie Habagishi and Shoko Kasuga analyzed the data. Chie Habagishi, Shoko Kasuga, Yohei Otaka, Meigen Liu, and Junichi Ushiba wrote the paper. Shoko Kasuga, Chie Habagishi, and Junichi Ushiba revised the paper. Final version of the manuscript was approved by Junichi Ushiba.

### Conflict of interest statement

The authors declare that the research was conducted in the absence of any commercial or financial relationships that could be construed as a potential conflict of interest.
